# Assessing and managing freshwater ecosystems vulnerable to environmental change

**DOI:** 10.1007/s13280-014-0566-z

**Published:** 2014-11-15

**Authors:** David G. Angeler, Craig R. Allen, Hannah E. Birgé, Stina Drakare, Brendan G. McKie, Richard K. Johnson

**Affiliations:** 1 Department of Aquatic Sciences and Assessment, Swedish University of Agricultural Sciences, Box 7050, 750 07 Uppsala, Sweden; 2 U.S. Geological Survey, Nebraska Cooperative Fish and Wildlife Research Unit, School of Natural Resources, University of Nebraska–Lincoln, 101 Hardin Hall, 3310 Holdrege Street, Lincoln, NE 68583-091 USA; 3 School of Natural Resources, University of Nebraska–Lincoln, 101 Hardin Hall, 3310 Holdrege Street, Lincoln, NE 68583-091 USA

**Keywords:** Global change, Resilience, Regime shifts, Monitoring, Management, Vulnerability

## Abstract

Freshwater ecosystems are important for global biodiversity and provide essential ecosystem services. There is consensus in the scientific literature that freshwater ecosystems are vulnerable to the impacts of environmental change, which may trigger irreversible regime shifts upon which biodiversity and ecosystem services may be lost. There are profound uncertainties regarding the management and assessment of the vulnerability of freshwater ecosystems to environmental change. Quantitative approaches are needed to reduce this uncertainty. We describe available statistical and modeling approaches along with case studies that demonstrate how resilience theory can be applied to aid decision-making in natural resources management. We highlight especially how long-term monitoring efforts combined with ecological theory can provide a novel nexus between ecological impact assessment and management, and the quantification of systemic vulnerability and thus the resilience of ecosystems to environmental change.

## Introduction

Freshwater ecosystems, including streams, rivers, lakes, riparian areas, and other wetlands, are highly vulnerable to stressors such as eutrophication, species invasion, land-use change, and increasing temperatures (e.g. Firth and Fisher 1992; Poff et al. 2002; Glen 2010; Boon and Raven 2012; Capon et al. 2013). Our understanding of the *vulnerability* (see definition of terms in italics in Box 1) of aquatic ecosystems is based on an extensive body of research, which provides insight into ecological responses, such as altered patterns in host–parasite interactions (Marcogliese 2001; Paull et al. 2012), body size structure (Yvon-Durocher et al. 2011), and food webs (Meerhoff et al. 2012; Shurin et al. 2012; Ledger et al. 2013). Studies also highlight a predicament long recognized by managers and researchers alike: (1) ecological responses to change are highly uncertain, and (2) gross generalization and prediction of the impacts of environmental change on freshwater ecosystems is impossible (e.g. Wilby et al. 2010).

**Box 1:** Glossary of terms used in the article

Term	Description
Vulnerability	*Species*-*level vulnerability* reflects a mismatch between functional traits of a species and their abiotic and biotic environment; for instance, when cold-stenothermic taxa are unable to cope with increasing thermal stress. *Systemic vulnerability* reflects conditions where the resilience of an ecosystem erodes, likely due to the loss of species that carry out critical ecosystem processes. Systemic vulnerabilities indicate the propensity of an ecosystem to undergo an undesired regime shift and/or have reduced ecosystem service provisioning capacity.
Regime shifts	Inherent to the ecological resilience definition is that ecological systems can undergo non-linear change or shift between alternative states, such as e.g. shallow lakes that show clear-water and turbid alternative states.
Resilience	*Ecological resilience* is a measure of the amount of change needed to transform an ecosystem from one set of processes and structures to a different set. An ecosystem with high resilience would require a substantial amount of energy to transform, whereas a low resilience system would transform with a relatively small amount of energy. *Engineering resilience* focuses on the recovery time of structural and functional ecosystem settings to pre-disturbance conditions, with a fast and slow return time indicating high and low engineering resilience, respectively.
Alternative stable state	An alternative stable state is defined by stable structures, functions, processes and feedbacks.
Adaptive capacity	Adaptive capacity is related to genetic and biological diversity, which provide ecosystems with the ability to maintain critical functions and processes during changing and/or novel environmental conditions.
Threshold	When an ecosystem crosses a threshold or tipping point its capacity to adapt to and cope with disturbances has been exhausted, and it abruptly reorganizes in a new regime with new structures, functions, and processes.
Functional trait	An individual-level characteristic that determines the role of a species on ecosystem processes (e.g. leaf litter decomposition) and its response to environmental factors.

Stress associated with environmental change can cause non-linear, rapid transitions between ecosystem states (i.e., *regime shifts*; Scheffer and Carpenter 2003). In fact, worst-case scenarios depict an erosion of *resilience* of freshwater ecosystems, facilitating undesired regime shifts (Meerhoff et al. 2012) with uncertain outcomes regarding the provisioning of ecosystem services in the future. Although there exist some efforts to identify and monitor warning indicators of regime shifts in ecosystems (e.g. Carpenter et al. 2011; Seekell et al. 2012; Veraart et al. 2012), the aforementioned uncertainty and lack of generalization across ecosystems make this approach difficult to develop (Hughes et al. 2013) and implement (Biggs et al. 2009). This uncertainty arises partly because, at least in the freshwater context, it is unclear how generalized regime shifts are across ecosystem types. Uncertainty also arises due to complex ecological responses that environmental change triggers in ecosystems. Interacting effects of climatic change and other, non-climatic, anthropogenic factors such as pollution, habitat fragmentation, and species invasions, are often highly context dependent (Covich et al. 2004; Gillson et al. 2013), causing synergistic or antagonistic ecological responses. Biogeographical, altitudinal, and climatic contexts may further modulate or drive outcomes. This limits our ability to infer general patterns of freshwater ecological responses to environmental change at a scale commensurate with management decision-making.

To determine whether freshwater ecosystems will experience future regime shifts in response to environmental change, researchers and managers must strategically parse limited resources for better research, management, and conservation of aquatic ecosystems. Thus, robust tools are needed to reduce the uncertainties related to vulnerability assessment of freshwater ecosystems. In this paper, we seek to provide a first step to accomplish this by providing an overview and application of statistical and modeling methods that allow for quantification of the systemic vulnerabilities and thus the resilience of freshwater aquatic ecosystems to environmental change. We show how long-term monitoring, combined with other approaches, can be used to achieve these goals. Specifically, we highlight a novel nexus between long-term monitoring efforts, resilience, and ecological theory. Combining monitoring and theory can provide new insight for refining ecological impact assessment. Also, resource use in environmental management can be improved through a better mechanistic understanding of the ecological complexity that is inherent to ecosystems.

## Assessing vulnerability

We discuss a framework that may provide researchers and managers with tools to reduce the inherent uncertainty of vulnerability assessments without sacrificing the complexity needed to understand ecosystem structures and processes. We frame this discussion in the context of ecological resilience, which describes the capacity of a system to absorb disruption without moving to an *alternative stable state*. Resilience theory is useful because it attempts to quantify characteristics of ecological complexity, thus, allowing for an assessment of critical ecosystem attributes that determine the system’s capacity to cope with disturbances.

## Resilience theory in a nutshell

The term resilience means different things in different contexts. Engineering resilience takes on the commonly understood definition of the ability and time required to “bounce back”, like a rubber band bending under force, but snapping back to its initial shape once the force is removed. This type of resilience has been applied in aquatic systems (Gaudes et al. 2010; Gerisch et al. 2012; Robinson 2012), but tells us little about the system’s *adaptive capacity*. Because engineering resilience depends on the rubber band’s initial strength and plasticity, it fails to account for adaptation in the face of change; that is, the rubber band does not “learn” from the force.

Ecological resilience has also been applied to understanding freshwater ecosystems (e.g. Bogan and Lytle 2011; Ireland et al. 2012; Angeler et al. 2013a), and emphasizes the ability of a system to absorb disturbance and its ability to “learn” and adapt to disturbances through mechanisms such as natural selection, plastic physiological response, and feedback loops. Thus, the ecological definition of resilience is much more well-suited to studying the systemic vulnerabilities of ecosystems to environmental change than the engineering definition of resilience. More specifically, Holling (1973) defined ecological resilience as a measure of the amount of change or disruption that is required to transform a system from being maintained by one set of reinforcing processes and structures to being maintained by a different set of processes and structures. Inherent to this definition is that ecological systems can undergo non-linear change or shift between alternative states (i.e. regime shifts). Ecosystems can operate in multiple basins of attraction, and therefore, do not have an equilibrium regime. The following example makes the difference between engineering and ecological resilience clear.

It is recognized that environmental change will likely trigger more frequent non-linear changes (regime shifts) in aquatic ecosystems (Meerhoff et al. 2012). Shallow lakes are well-known models of such shifts: upon excessive nutrient enrichment, lakes shift from a clear-water state dominated by submerged macrophytes (desired state) to a state characterized by turbid water, frequent algal blooms that are often toxic, and reduced ecosystem service provisioning in the degraded or undesired state (Carpenter and Cottingham 1997; Scheffer 1997). Both states are stable, in that a high level of intervention is needed to disrupt the mechanisms that maintain the definitive system structure and function of the degraded state. When a *threshold* of disturbance is reached, the mechanisms of the desired state are reorganized with a new set of feedbacks and mechanisms; a process even intensive management intervention is unlikely to reverse. Engineering resilience does not account for alternative stable states, and incorrectly implies that an undesirable state would inevitably revert to a desired state without management interaction given enough time.

Ecological resilience is broader than the often-used concept of “stability” because it explicitly considers a compartmentalization of ecological structures and processes by scales that are commensurate in space and time (Holling 1992; Angeler et al. 2013b; Allen et al. 2014). For example, at the individual zooplankton scale range, predation and competition occur in space and time at cm^3^ to m^3^ and hours to days, respectively, in the context of a lake that ranges with surface areas from multiple m^2^ to km^2^ and water renewal times lasting years to decades, in a landscape that scales hundreds to thousands of km^2^ and has formed over centuries and millennia. This multi-scale spatiotemporal consideration of ecological resilience is useful because the impacts of environmental change differ greatly depending on the scale of observations (Angeler et al. 2011; Nash et al. 2014). Thus, ecological resilience provides a framework with which to identify both the type and magnitude of ecological disturbance across spatial and temporal ecological scales. This explicit view of scaling relationships in ecological systems permits quantifying several mutually non-exclusive core concepts and issues that are thought to confer system resilience. These core concepts are briefly outlined below.

### Core concepts

Essential to the understanding of the following key concepts is the notion that ecosystem processes (e.g. flux of matter and energy, primary productivity) depend on functional attributes of species within ecosystems, and species’ responses to disturbances. This is a subtle but important departure from the idea that ecosystem processes are mostly reliant on structural community attributes, like species richness (Hooper and Vitousek 1997; Nyström 2006; Mori et al. 2013). Explicit to the systemic assessment of vulnerabilities is the quantification of the distributions of *functional traits* at multiple scales of space and time. Understanding how traits are distributed within and across scales has implications for the resilience of ecosystems.

#### Cross-scale resilience, functional redundancy, and the insurance effect

Peterson et al. (1998) described the cross-scale resilience model that proposes that the resilience of ecological processes, and ultimately ecosystems, depends in part on the distribution of functional traits of species within and across scales of space and time. Within a given scale, resilience increases due to an overlap of functional traits among species of different functional groups that operate at the same scales (Allen et al. 2005). The recognition that an increase of resilience is due to an overlap of functions within scales relates to the concepts of functional redundancy, or the “insurance hypothesis” (e.g., Yachi and Loreau 1999; Mori et al. 2013). These concepts received significant research attention in an effort to better elucidate the relationships between biodiversity and ecosystem functioning (BEF). However, much of that research neglects the fact that ecological processes are compartmentalized by scale. Thus, combining BEF approaches with the cross-scale resilience model may yield a more mechanistic understanding of biodiversity and its role in ecosystems and management.

#### Response diversity

The concept of response diversity (Elmqvist et al. 2003) is useful for disentangling the effect of within and cross-scale species distributions on resilience. Rather than focusing on the redundancy of a specific functional trait across scales, this concept emphasizes the variation in responses to environmental change by species within a functional group within scales. In other words, response diversity considers the functional make up of a species accounting for multiple traits (Mori et al. 2013) that modulate species responses through, for instance, distinct colonization, growth, competition, and dispersal abilities. If, for example, a community includes multiple species comprising a single functional group, and all members of that functional group have similar trait configurations and interact with their environment at the same scale, it can be expected that all respond similarly to disturbance. In this case, response diversity, and therefore resilience, is low, meaning that an entire functional group responds in the same way to a disturbance event, and all are truly redundant. Thus, the ability to quantify response diversity within and across scales of ecological systems would provide further insight into their relative resilience to environmental change. However, the expression of functional traits can vary according to abiotic and biotic context (McKie et al. 2008). For instance, species interactions might suppress or strengthen expression of some traits, as may particular environments. This suggests that response diversity needs to be scrutinized as a function of the variability of trait expression.

#### The role of rare species

In ecological systems, most species are rare. In other words, most species are represented by only a small number of individuals and/or are restricted to selected habitats. However, the role of rare species to system resilience, and their vulnerability to environmental change remains unclear. Mouillot et al. (2013) recently argued that distinct combinations of functional traits are supported predominantly by rare species in coral reefs, alpine meadows, and tropical forests. They concluded that a loss of these rare species, even within highly diverse systems, could have disproportionately negative effects on ecosystem functions.

There is also evidence that rare species may actually replace dominant species following disturbance, contributing to the continued existence of an ecosystem in its desired stable state (Walker et al. 1999). This suggests that rare species likely contribute an important but somewhat unpredictable level of adaptive capacity to the system. It is clear that inference about the vulnerability of ecosystems to environmental change can be improved when accounting for abundance patterns in the analysis.

In some resilience assessment methods (i.e., discontinuities in animal body size; Allen and Holling 2008), species dominance patterns, and therefore the role of both rare and dominant species, are not accounted for. However, the importance of uncommon and common species, and their relevance for resilience, can be scrutinized in explicit time series (Baho et al. 2014) and spatial modeling (Göthe et al. 2014).

## From theory to measurement

The cross-scale resilience model highlights the need to identify and define the scales of structure present in a system. There are several methods available to infer scale-specific patterns in ecological systems, but these methods differ in their assumptions, which is an important consideration when inferring resilience and comparing results based on different methods. Pros and cons of various methods described below are summarized in Table 1.

**Table 1:** Comparison of methods available for assessing cross-scale structures necessary for studying systemic vulnerabilities to global change

Method	Data sets	Advantages	Limitations
Discontinuity analyses (GRI, CA, CART, BCART, KDE)	Univariate, rank-ordered, log-transformed data (e.g., body size or mass)	Data easy to obtain either from available sources or through measurement	Species dominance patterns not explicitly accounted for
		Simple assessment of non-linear (scale-specific) structures in data	Resilience assessment limited to the evaluation of cross-scale patterns
			Limiting assessment of ultimate factors causing discontinuities
Time series and spatial modeling (Canonical ordinations^a,b^; wavelet analyses^c^)	Multivariate; species abundance, biomass and/or presence–absence data	Species abundances accounted for	Data acquisition labor intensive, high resource demand
		Separating the role of dominant and rare species	Higher analytical complexity relative to discontinuity analysis
		Evaluation of complementary aspects of resilience and adaptive capacity	Scales and patterns of structure contingent on sampling frequency and length
		Relating patterns to dynamic environmental change	Limited availability of adequate long-term data

*GRI* gap rarity index, *CA* cluster analysis, *CART* classification and regression trees, *BCART* Bayesian CART, *KDE* Kernel density estimates (see text)

^a^Angeler et al. (2009), an example for time series modeling

^b^Dray et al. (2006), showing the modeling framework for assessing spatial resilience

^c^Keitt and Fischer (2006), time series modeling

Classification and regression tree analyses and their Bayesian implementations (Chipman et al. 1998), kernel density estimation (Havlicek and Carpenter 2001), and the gap rarity index (Restrepo et al. 1997) have all been used to evaluate discontinuities in animal body mass distributions. The underlying assumption is that the discontinuous organization of ecological systems is mirrored in the structure of animal communities. Holling (1992) posited that behavioral, life history, and morphological attributes of animals adapt to discontinuous environmental patterns because these patterns reflect opportunities for food, shelter, and other resources. Indeed, Holling (1992) found a correlation between breaks in distributions of animal body mass, an integrative variable allometric with many physiological and ecological attributes (Peters 1983), and discontinuities in structures and processes in the boreal forest of Canada. He interpreted aggregations of species (or modes) along body mass distributions as scales at which resources and structure are available to organisms that have evolved to exploit resources at these specific scales but not at other scales. In contrast, gaps (discontinuities or troughs) in the distribution reflect the transition between structuring processes, and thus scaling regimes (i.e. thresholds). At these transitions, there is no ecological structure or resource pattern with which animals can interact, or there is great variance and instability in the structures or patterns (Allen and Holling 2008).

Discontinuity analyses are effective for identifying the number of dominant scales present in animal communities or other complex systems (Allen et al. 2005; Allen and Holling 2008; Nash et al. 2014). However, while body mass is an important trait of animal species, the lack of body mass data for other taxonomic kingdoms (e.g. fungi, plants) has led to a research bias towards animals in discontinuity analyses. Also, because body mass integrates processes acting at distinct evolutionary and ecological time scales, our ability to discern among the relative importance of ultimate factors generating discontinuous body mass distributions is limited. Furthermore, species abundances are not accounted for in discontinuity analyses of body mass. This analysis, therefore cannot distinguish between the role of dominant versus rare species (Table 1). Using data independent of body mass, such as population variability, to identify scaling patterns may increase the robustness of discontinuity analyses (Table 1).

Ecosystems are generally measured and managed at scales tractable to humans, extending between tens to thousands of meters and ranging from weeks to a few decades. Time series modeling allows us to identify the scales of temporal frequencies in complex systems, and makes it possible to track the imprints of environmental change over time (Keitt and Fischer 2006; Angeler et al. 2011, 2013c). For example, analysis of long-term data has revealed discrete groups of species that exhibit distinct temporal frequencies, with some responding to slow environmental variables and others responding to fast variables (e.g., Angeler et al. 2013c). Multi-scale hierarchical spatial modeling (e.g. Dray et al. 2006) allows for the extension of resilience assessments from ecosystem to landscape scales or ecological networks (Göthe et al. 2014), providing opportunities to test the vulnerability of entire networks of ecosystems or regional landscapes to environmental change (Cumming 2011). Both time series and spatial modeling hold much promise, but the scales of patterns and structure that can be discerned have upper bounds set by the limit of the temporal extent or number of sites covered in the data series, and lower bounds set by the temporal frequency or spatial resolution of sample collection. The following case studies show the usefulness of discontinuity analysis and time series modeling for systemic vulnerability assessments to environmental change. The first two case studies are based on long-term monitoring data from the Swedish National lake monitoring program (Fölster et al. 2014), which highlight the usefulness of monitoring efforts to assess the systemic vulnerability and thus the resilience of ecosystems in the face of environmental change. The third case study from the Everglades demonstrates a complementary approach, and shows how resilience can be quantified using discontinuity analyses when long-term monitoring data are lacking.

## Case studies

### Subarctic lakes in Sweden

Ecosystems at high altitudes and latitudes are likely to be especially vulnerable to the effects of environmental change (Wrona et al. 2006). Angeler et al. (2013c) assessed the responses of littoral invertebrate communities to changing abiotic conditions in subarctic Swedish lakes with long-term data (1988–2010) from the Swedish monitoring program of surface waters. They compared the responses with those of more southern, hemiboreal lakes. Using multivariate time series modeling to identify dominant and distinct temporal frequencies in the data, the authors tracked community changes at distinct temporal scales. They then determined the distribution of functional feeding groups of invertebrates within and across temporal scales, evaluating resilience based on the predictions made by the cross-scale resilience model by Peterson et al. (1998).

The authors identified two distinct patterns of temporal change within the invertebrate communities across the lakes. The first pattern was one of monotonic change associated with changing abiotic lake conditions due to environmental change-mediated impacts on water clarity. The second pattern showed fluctuations largely unrelated to gradual environmental change. Thus, two dominant and distinct temporal frequencies (temporal scales) were present in all analyzed lakes. While the scale-specific distribution of individual feeding groups varied between subarctic and hemiboreal lakes, they shared overall similar functional attributes (e.g. evenness, diversity). The functional redundancy within and among the observed temporal scales was similar across lakes, highlighting the similarity in resilience characteristics across both subarctic and hemiboreal lakes. Another important finding from this case study was that cold-stenothermic species have been lost and replaced with warm-tolerant species in the subarctic lakes. However, this did not yield any observable loss in the resilience of subarctic lakes. Thus, the functional compensation of feeding group attributes over time, despite structural community change, currently seems to maintain the functional underpinnings of ecosystem processes, conferring robustness to subarctic lakes.

### Acidified Swedish lakes

The subarctic lakes study aimed at identifying resilience characteristics between lake types that have potentially different vulnerabilities to environmental change (that is, without knowing a priori how human action has affected these lakes). However, many cases exist where humans have already had a negative effect on ecosystems. Such a case is anthropogenic acidification, leading to biodiversity loss in many lakes that were sensitive to acidification due to their low acid buffering capacity. There is evidence that acidification caused a regime shift in many Scandinavian lakes. Despite the implementation of international policy to mitigate the impact of acidification, many lakes have shown weak chemical and biological recovery and thus resisted returning to previous conditions (Johnson and Angeler 2010; Angeler and Johnson 2012).

**Fig. 1:**
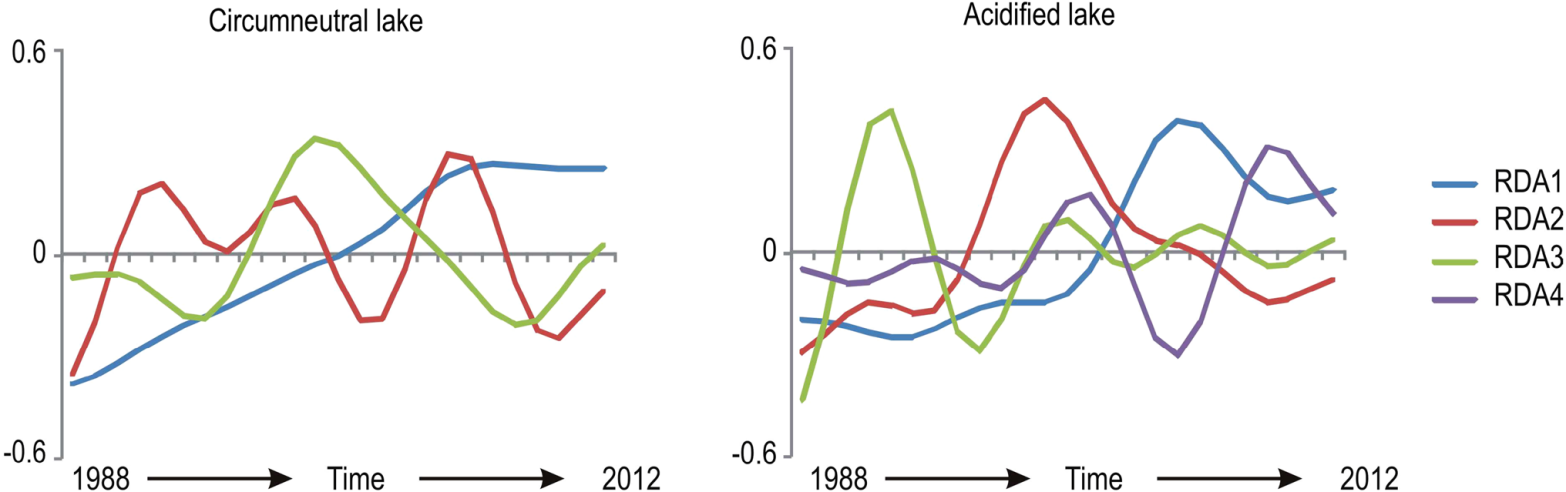
Example of time series modeling showing temporal patterns of species groups associated with canonical (RDA) axes in one circumneutral and one acidified lake. Shown are the temporal patterns with 3 and 4 significant canonical axes in the time series models, respectively, that capture the temporal scaling structure in the data

Similar to the subarctic lakes study, Angeler, Allen, and Johnson (unpublished results) compared littoral invertebrate communities to changing abiotic conditions in acidified (degraded state) and circumneutral (desired, undegraded state) Swedish lakes with long-term data (1988–2012), using the time series modeling approach described above. They again identified dominant and distinct temporal frequencies in the data, which in the time series models are associated with different canonical (RDA) axes (Fig. 1). That is, these canonical axes represent groups of species with distinct fluctuation patterns. In addition to dominant temporal frequency patterns (or scales), they assessed species with stochastic dynamics that were not associated with the temporal frequency patterns observed, and that presumably comprise rare species without clear temporal patterns. They determined the distribution of functional feeding groups of invertebrates within and across temporal scales, and in the stochastic group of species. Three patterns of temporal change within the invertebrate communities were identified that were consistent across the lakes. The first pattern (canonical axis 1) comprised species that showed monotonic change associated with changing abiotic lake conditions (blue lines in Fig. 1). The second and third patterns associated with canonical axes 2 and 3, respectively, showed fluctuation patterns of invertebrate species groups largely unrelated to gradual environmental change (red and green lines; Fig. 1). Thus, at least three distinct temporal frequencies (temporal scales) were present in all lakes analyzed. As was the case in the subarctic lakes study, acidified and circumneutral lakes shared overall similar functional richness, evenness, diversity, as well as similar redundancy patterns within and across the observed temporal scales and in the stochastic species group. Again, these similar resilience characteristics highlight similar systemic vulnerabilities to environmental change among lakes. That is, although acidified lakes have already undergone a potential regime shift the results suggest that these lakes have a similar likelihood to circumneutral lakes of undergoing further regime shifts if there is ongoing environmental change. It also highlights that the acidified lakes unlikely will return to a non-acidified ecological state without management aimed at breaking the feedbacks that maintain the acidified state.

These similar resilience characteristics observed in both lake types have been attributed to functional compensation processes, which have been shown in acidified lakes (Klug et al. 2000; Fischer et al. 2001). Although richness of acid-sensitive taxa was lower in the acidified lakes relative to the circumneutral lakes in this study, overall taxon richness was only marginally higher in circumneutral lakes. This suggests that other species, tolerant to acidification stress have likely substituted acid-sensitive taxa (Layer et al. 2010) and compensated for the loss of functions of these sensitive taxa.

The finding of similar vulnerability patterns between acidified and circumneutral lakes are encouraging, because although acidified lakes in a degraded state often have higher aluminum toxicity and damaged fish communities, limiting their value for fisheries, they have often clearer waters, contributing to other recreational (boating) and esthetic services. Some of these services might be at stake if acidified lakes are more vulnerable to further regime shifts with environmental change.

Both case studies make clear how long-term monitoring efforts, combined with an ecological complexity approach that is often neglected in assessing environmental change problems, can facilitate an evaluation of systemic vulnerability. However, long-term monitoring is lacking for most ecosystems. The next case study shows an alternative approach to quantify resilience with limited data.

### The Florida Everglades

Using the vertebrate fauna of the Everglades wetland complex of south Florida (USA), Forys and Allen (2002) quantified how the loss of native amphibian, bird, reptile, and mammal species concurrent with invasions by non-native taxa altered functional group richness within and across ecosystem scales. They carried out discontinuity analyses on rank-ordered body mass data to identify groups of species that operate in similar scaling regimes. They found that despite large changes in species composition due to local extinctions and successful invasions, functional group richness did not change significantly within scales. There was also no significant loss of overall redundancy of functional traits across scales, and the overall body mass pattern did not undergo substantial change as a result of invasions. This highlights the robustness of the underlying relationships between structure and processes regardless of species identity, and the broader resilience of these communities to the surplus of anthropogenic stressors that currently affect the Everglades.

## A conceptual model for measuring systemic vulnerabilities to environmental change

**Fig. 2:**
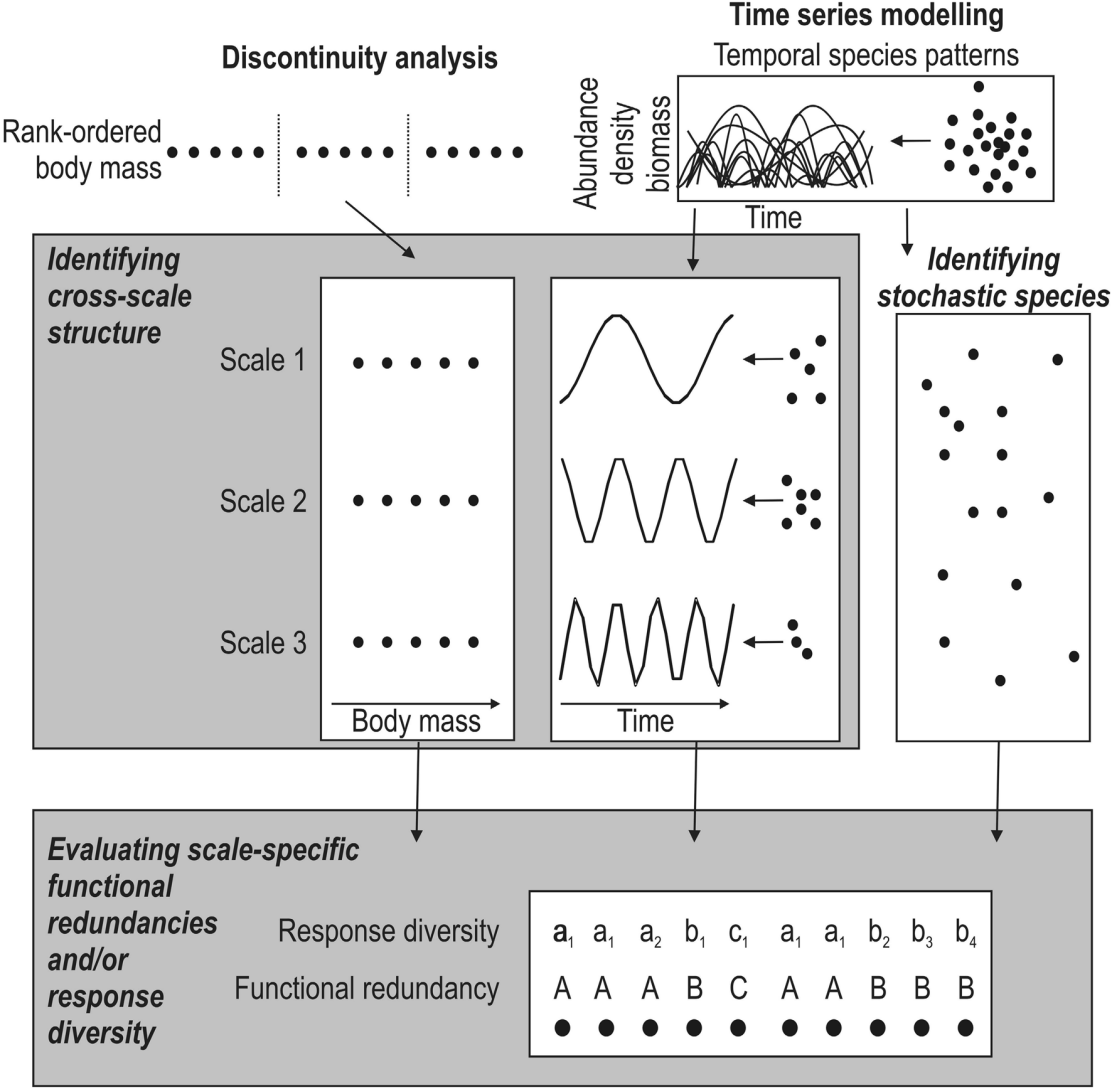
Conceptual model outlining approaches for identifying scale-specific structures necessary for understanding the systemic vulnerability of ecological systems to global change. In a first step, discontinuity analysis or time series analysis can be used to identify the cross-scale structure in data sets; time series analyses also allow the identification of species with stochastic patterns that are not contributing to cross-scale structure. After identifying cross-scale (and stochastic) patterns, functional redundancy, and response diversity can be assessed for species explaining scaling patterns and also stochastic species

By combining the case studies with our understanding of resilience theory, we present a conceptual model to empirically assess systemic vulnerability of freshwater ecosystems to environmental change (Fig. 2). Our model builds on discontinuity analysis and time series modeling based on long-term monitoring, both proven useful for assessing resilience. These techniques are already employed by several resilience assessment studies (overview in Allen et al. 2014; Nash et al. 2014), facilitating comparisons across communities and ecosystems. Time series modeling is based on canonical ordinations using redundancy analysis (Angeler et al. 2009), which is a temporal analog to multi-scale spatial modeling (Dray et al. 2006). Thus, for simplicity we only show time series modeling in Fig. 2. It is beyond the scope of this paper to present the methodological details, which can be found in Allen et al. (2005), Allen and Holling (2008) (discontinuity analysis), Angeler et al. (2009), and Angeler et al. 2013a (time series analysis).

Our conceptual model is novel in that it emphasizes the need to identify the scale-inherent structures in data sets for assessing systemic vulnerabilities (Fig. 2). It especially shows how temporal scaling patterns can be made explicit when long-term monitoring data are available. Once the scaling patterns have been identified, taxa can be associated with these scales, and their contributions to within- and cross-scale redundancies evaluated. If multiple functional traits are identifiable that allow for the estimation of potential responses to disturbance, the functional redundancy analysis can be refined with an assessment of response diversity patterns compartmentalized by scale. The model shows how complexity attributes of ecological systems can be evaluated in two straightforward steps to better understand systemic vulnerability to environmental change and the resilience of ecosystems.

### Application to management

Resources for managing ecosystems are always limited, requiring the identification of trade-offs and priorities. Freshwater ecologists and managers are challenged to identify and protect ecosystems that provide rare and vital services but are vulnerable to regime shifts. Our conceptual model provides a starting point for reducing uncertainty by identifying systems that are vulnerable to environmental change, and allowing for standardized comparative analyses of systemic vulnerabilities within and across ecosystems. We believe this approach will facilitate the efficient and effective identification of ecosystems requiring management priority. By quantifying and comparing scaling patterns and the distribution of functional traits within and across scales, inference about the relative resilience of freshwater ecosystems can be made.

**Fig. 3:**
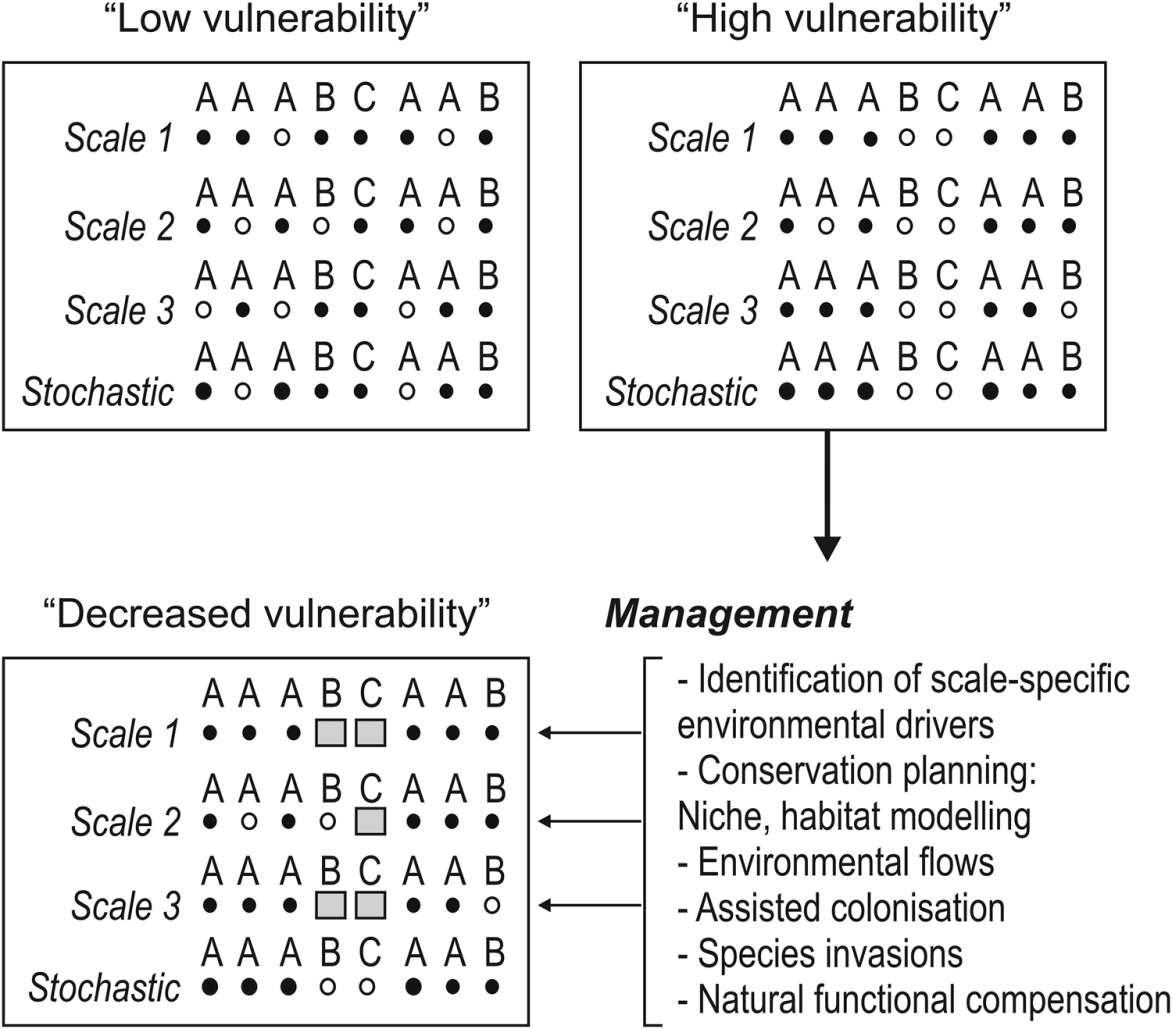
Scenarios contrasting high and low systemic vulnerabilities to environmental change of ecological systems, and how vulnerability can be decreased through management. The “low vulnerability” scenario shows that functions *A*, *B*, and *C* are carried out by “vulnerable” (*white dots*) and “tolerant” (*black* *dots*) species and all functions are redundant within and across scales. In the “high vulnerability” scenario within- and cross-scale redundancies of functions *B* are decreased, and function *C* has been lost. The model shows how management can be geared towards maintenance of these functions

We illustrate this with the following hypothetical scenarios (Fig. 3). In these scenarios we incorporate species vulnerabilities, accounting for their physiological sensitivities to stressors such as increasing temperatures that might contribute to their extinctions. While a host of direct and indirect traits contribute to adaptive capacity in the face of disturbances, we selected thermal traits as the focus of our model. Sensitive species are symbolized by the white dots and distinguished from species with higher tolerances to environmental stress (black dots) in our scenarios (Fig. 3). In the “low vulnerability” scenario, species within a community carry out the hypothetical functions A, B, and C. In this “low vulnerability” scenario, function A has the highest within- and cross-scale redundancy, followed by functions B and C. All functions are carried out by “vulnerable” and “tolerant” species. Ignoring possible functional compensation processes, this scenario suggests that an extinction of vulnerable species is less detrimental for the ecosystem, because all functions are still carried out by tolerant species, both within and across scales, once sensitive species go extinct. If we simply reshuffle the vulnerability characteristics of species, we can obtain a contrasting scenario that reflects a high systemic vulnerability to environmental change. In this scenario, extinctions may decrease the within- and cross-scale redundancies of function B, and lead to a loss of function C altogether. This reveals that the system’s capacity to fulfill critical processes is associated with its functions. In turn, functions that are imperative for the provisioning of essential ecosystem services are jeopardized. If managers can identify ecosystems with such vulnerability characteristics, management priorities can be geared towards maintenance of these functions (Fig. 3).

It is not our aim to provide an exhaustive list of how our model could inform management; environmental change can have context-dependent effects and will therefore require site-specific approaches. However, the following considerations can provide guidance for tailoring structured and site-specific management plans within the context of a growing understanding of general ecosystem response to various aspects of environmental change.

It is increasingly clear that environmental change has scale-specific impacts (Nash et al. 2014). Our scenarios emphasize the need to identify scales amenable to management. For example, species that operate in scaling regimes within very broad spatial (e.g. global) and temporal (e.g. centuries, millennia) extents may be more difficult, if not impossible, to manage. Case studies 1 and 2 make clear how the effects of environmental change can be particularly strong at scales with slow biotic and abiotic system dynamics operating over broad spatial extents. Similarly, our current governance structures lack the design, capacity, and resources available to cope with environmental change management of freshwater ecosystems at such scales (Nilsson and Persson 2012). It is therefore necessary to identify scales that are either unaffected by environmental change or that allow management of scales tractable by current natural resources governance schemes. Managing at these scales can maintain or increase functional ecosystem properties and avoid undesired regime shifts.

In practice, this means that management first needs to identify spatial and temporal scales in ecosystems that might be the most effectively managed. Our model (Fig. 2) shows how this can be achieved in objective ways using the quantitative approaches outlined in this paper, rather than arbitrary and researcher-based definition of scales that may muddle ecosystem-inherent patterns and processes. At any of these identified scales, management can target, maintain or increase functional redundancy through, for instance, assisted translocations (Olden et al. 2011) to compensate for a potential loss of redundancies at unmanageable scales. Detailed spatial and temporal conservation planning (Hermoso et al. 2012) and other niche (Pearson and Dawson 2003) and habitat modeling (Keith et al. 2008) can be very useful to manage the abiotic habitat template (e.g., environmental flows; Arthington et al. 2010; Yen et al. 2013) to optimize the viability of resident species and assisted colonizers at these scales (species symbolized with gray squares in Fig. 3). Optimizing assisted colonization may be desirable if maintenance of local functions through natural colonization processes from regional sources is limited (Thompson and Shurin 2012). The role of non-native species, while under debate, still merits our attention. Non-native species have the potential to compensate for the loss of functions and increase the resilience in ecosystems, thereby decreasing whole ecosystem vulnerability to environmental change. It is critical to note, however, that the benefits of “assisted invasions” must be carefully designed and balanced against the documented deleterious effects of invasions on freshwater ecosystems.

## Conclusion and future challenges

Both researchers and managers are in need of applicable, effective tools to understand freshwater ecosystem vulnerability to environmental change. Our conceptual model provides a first step in this direction. The model provides opportunities to compare vulnerability and resilience across ecosystems in relative terms; that is, specific ecosystems with high vulnerability can be identified through comparative assessments, helping to set management priorities. Our model limits an assessment of vulnerability and resilience of individual ecosystems in absolute terms. This means that data before and after regime shifts are needed to assess when the resilience of ecosystems begins to erode, and the risk of a regime shift increases. However, transitions between regimes can be slow, unfolding over centuries and millennia (Spanbauer et al. 2014), limiting decision-making at scales commensurate with current management schemes. Further, it is critical to note that future responses are not accounted for in our assessment process. This means that ecosystems that currently appear to be resilient like the subarctic lakes (case study 1) or the Everglades (case study 3) may face an erosion of resilience in the future (Forys and Allen 2002).

Further testing of our model across ecosystems holds the potential to assist managers in prioritizing ecosystems from management actions. Our model holds the potential to reduce uncertainty associated with environmental change vulnerability assessments; it also supports a novel approach to freshwater ecosystem management. It is clear that a systemic assessment of environmental change vulnerabilities requires a great amount of data of sufficient temporal span and spatial extent. Exceptional data sets from long-term monitoring programs have proven very useful so far, but the broader application of promising temporal or spatial modeling tools is currently limited by the general lack of standardized, long-term (centuries, millennia) data with good spatial resolution. Management must continue to emphasize long-term monitoring efforts (Maberly and Elliott 2012; Vihervaara et al. 2013), which, in combination with paleontological data, may allow for a better understanding of complex system responses to environmental change.

Additionally, some level of monitoring must occur in concert with the application of our conceptual model to create an iterative approach capable of capturing ecological complexity and variability over time. Fortunately, existing data do allow us to empirically study vulnerability patterns in ecosystems. These, in combination with specifically designed experiments (Ledger et al. 2012), provide opportunities for obtaining complementary and more mechanistic information between ecosystem structure and process. Improved trait-based information will further strengthen inference, especially when data can be divided to reveal trait response to disturbance (Sterk et al. 2013). Aquatic communities (microbes, plankton) are especially suitable for experimental manipulation, facilitating hypothesis testing about the influence of perturbations on ecosystems and their structural attributes and processes.

This paper demonstrates how long-term monitoring, combined with other approaches, can be used to create a nexus with ecological theory to refine ecological impact assessment and improve environmental management. A better mechanistic understanding of the ecological complexity that is inherent to ecosystems is needed to improve our knowledge of ecosystem responses to environmental change. This work shows how this complexity can be quantified through the use of monitoring data.
